# A critical review of current laboratory methods used to evaluate mosquito repellents

**DOI:** 10.3389/finsc.2024.1320138

**Published:** 2024-01-18

**Authors:** Hailey A. Luker

**Affiliations:** Molecular Vector Physiology Laboratory, Department of Biology, New Mexico State University, Las Cruces, NM, United States

**Keywords:** mosquito repellents, laboratory assays, spatial repellency, contact repellency, methods review, mosquito attractants, standardized methods, repellent efficacy

## Abstract

Pathogens transmitted by mosquitoes threaten human health around the globe. The use of effective mosquito repellents can protect individuals from contracting mosquito-borne diseases. Collecting evidence to confirm and quantify the effectiveness of a mosquito repellent is crucial and requires thorough standardized testing. There are multitudes of methods to test repellents that each have their own strengths and weaknesses. Determining which type of test to conduct can be challenging and the collection of currently used and standardized methods has changed over time. Some of these methods can be powerful to rapidly screen numerous putative repellent treatments. Other methods can test mosquito responses to specific treatments and measure either spatial or contact repellency. A subset of these methods uses live animals or human volunteers to test the repellency of treatments. Assays can greatly vary in their affordability and accessibility for researchers and/or may require additional methods to confirm results. Here I present a critical review that covers some of the most frequently used laboratory assays from the last two decades. I discuss the experimental designs and highlight some of the strengths and weaknesses of each type of method covered.

## 1 Introduction

Mosquito-borne diseases pose a massive threat to public health. Rising temperatures worldwide expand the geographical range of many key vector species, increasing the number of people at risk of contracting these diseases (1–5). Mosquitoes can transmit human pathogens that cause malaria, dengue, and West Nile, to name a few. Pathogen transmission occurs due to the blood-feeding constraint of anautogenous mosquitoes to complete their life cycle. When an infected mosquito takes a blood meal from a host, the host can become infected and vice versa (6–8). Integrated vector management (IVM) is an approach to mitigate pathogen transmission on a global scale (9–12). Some strategies of this approach, that target mosquito pathogen transmission, are controlling mosquito populations using pesticides, reducing larval habitat near human infrastructure, and educating communities on the mosquito life cycle and the risks that they pose (13–16). When used in tandem with others, a powerful strategy for individuals to implement regularly to prevent mosquito bites is the proper use of effective repellents (17–21).

Understanding the mode of action of mosquito repellents has been a large topic in the vector biology community for decades and has only been partially explained for some mosquito repellent active ingredients (22–28). The literature on this topic is vast and can easily fill several review papers, therefore I will only briefly touch on this topic (17, 29–34). To provide a general description, mosquito repellents target chemoreceptors associated with olfactory and/or gustatory organs, as well as other appendages that have chemoreceptive sensilla like the wings and tarsi (35–41). Mosquito repellents can act on chemoreceptors in various ways that elicit a repellent response in mosquitoes. Some of these ways include overstimulating or blocking specific chemoreceptors, or by masking odors (23, 42–46). In this review, I will be covering laboratory methods that test the behavior of mosquitoes in response to potentially repellent treatments.

There are two major categories of mosquito repellents commonly found in the literature (47–52):

- Spatial repellents.
- Contact repellents.

A mosquito repellent can convey either one type of repellency or both (27, 38, 53). Spatial repellents can be applied in several different forms including topical treatments like lotions and sprays or as devices that aerosolize repellent molecules into the proximal area. Spatial repellency is typically observed by the absence of mosquitoes in the vicinity or by the significant decrease in mosquitoes physically touching a treated object or individual (54, 55). The other category of repellency is contact repellency. Contact repellents repel mosquitoes that come into direct contact with the product and are usually applied topically through sprays or lotions. Contact repellency is typically observed when mosquitoes land on a treated host or object without proceeding to initiate feeding behaviors, like probing, and instead promptly fly away (41).

There are hundreds of commercially available mosquito repellent products available on the market worldwide (56–58). These products often contain active ingredients such as DEET (N,N-diethyl-meta-toluamide), Picaridin, IR3535, or para-menthane-diol (PMD) (59, 60). However, there is still a continuous search for effective alternatives to these products (61). One reason for this continued search is the general negative consumer opinion regarding the safety of the synthetically-derived active ingredients found in long-lasting mosquito repellent products (58, 62). This concern persists even with reports that conclude these active ingredients are safe when used as directed (63–70). The search for alternative products that do not utilize synthetic active ingredients is also amplified by the underlying premise that “natural” active ingredients are safer for human health and better for the environment (71).

Another reason for this continued search is because of individuals with skin allergies or sensitivities to some of the commonly-used active ingredients found in mosquito repellents (72, 73). It is challenging to formulate novel, effective mosquito repellents that can compete with the top-performing products on the market. New products should be scientifically tested for their repellent efficacy, before becoming commercially available, which can be done in a variety of ways (74, 75).

I present a literature review on common laboratory methods, from the last two decades, that can be used to measure the repellent efficacy of novel and established treatments on mosquitoes. For this review, the term “treatment” is defined as any material, chemical, or device that may elicit a response in mosquitoes.

## 2 Laboratory methods to test mosquito repellency

Laboratory assays used to measure the repellent efficacy of a specific treatment on mosquitoes vary greatly in:

- How repellency is measured.
• Repellency is typically determined by either recording changes in mosquito location, host-seeking behavior, or feeding behavior.
- The type of repellency being tested.
• Spatial or contact.
- The parameters of the experiment.
• Type of treatment being tested, dimensions of assay, presence of an attractant source, number of mosquitoes being tested, etc.

To organize this literature review, I divided laboratory assays that can be used to test the repellent efficacy of a treatment into two categories. Assays that measure mosquito behavior not related to host-seeking are categorized as “repellency assays without an attractant source”. Assays that measure mosquito movement and behavior during host-seeking by either incorporating a living host or a synthetic attractant are categorized as “repellency assays with an attractant source”. The assays that utilize attractant sources were further separated into two different groups: spatial repellency assays and contact repellency assays.

### 2.1 Repellency assays without an attractant source

These laboratory assays measure changes in mosquito behavior in the presence or absence of a treatment and are independent from mosquito host-seeking behavior. The assays covered in this category generally have the following common element in their experimental design: an apparatus that contains adult female mosquitoes that are monitored for changes in behavior. Mosquitoes can either fly towards or away from a treatment. Mosquito behavior in response to each treatment is recorded. Treatments that induce avoidance behaviors in mosquitoes, demonstrated by them relocating away from the treated area, are repellent.

The general strengths of these types of laboratory assays are that they can be easily reproduced and can rapidly screen many different treatments due to simple and straightforward experimental designs. Another strength is that these assays don’t involve mosquito host-seeking and feeding behavior. Mosquito feeding behaviors can vary widely between species and are influenced by variables like circadian rhythms, mosquito age, seasons, temperature, etc. (76). Assays that rely on mosquito feeding behaviors to test repellency require frequent control testing to assure mosquitoes are actively host-seeking during experiments. While not relying on mosquito feeding behavior to test repellency is a strength, it is also a weakness of these types of assays. A mosquito’s behavior in the presence of a treatment might be very different depending on if the mosquito is actively host-seeking or not. Additional tests using different types of assays are necessary to confirm if a treatment will actually protect humans from mosquito bites. Repellency assays without an attractant source are very useful, most notably in their ability to screen numerous treatments in a short amount of time and identify potentially effective mosquito repellents for a low cost and with relative ease.

Below I describe some common and useful laboratory assays that test the mosquito repellent activity of treatments in the absence of mosquito attractants (50, 77–91).

#### 2.1.1 Tube assays

**Figure 1:**
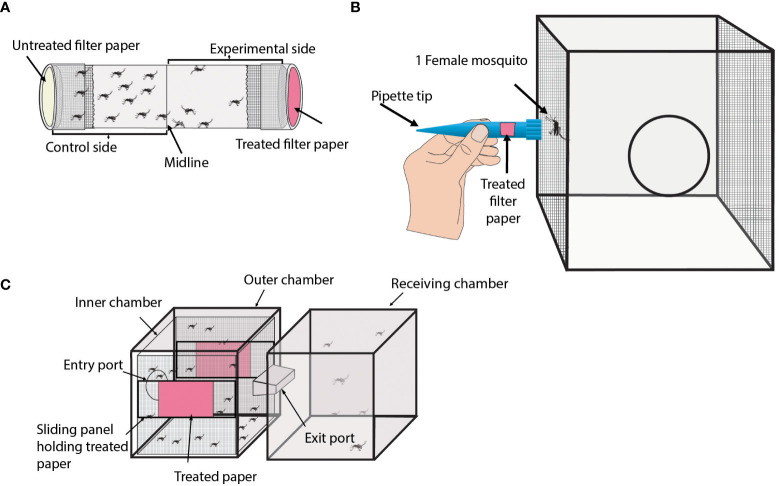
Laboratory repellency assays without an attractant source. **(A)** Diagram of the general format of a Tube assay. Shown is a transparent tube containing female mosquitoes. Both ends of the tube are capped to prevent mosquitoes from escaping the assay. The interior side of each cap contains a filter paper. The beige cap represents the untreated filter paper, and the pink cap represents the treated filter paper. **(B)** Diagram of the Close Proximity Response assay. Shown is a mesh-sided cage containing one female mosquito. A modified pipette tip containing a filter paper is shown being held up against the mesh region of the cage the mosquito is resting at. The pink box in the pipette tip represents a treated filter paper. **(C)** Diagram of the Excito-Repellency Test Chamber assay. Shown are two connected cages referred to as chambers. The left chamber is the main chamber and contains female mosquitoes. The main chamber has two treated papers shown in pink. The right chamber is the receiving chamber where repelled mosquitoes can relocate.

Overview: The Tube assay is a simple and low-cost technique that uses a hollow cylinder apparatus to measure mosquito behavior and location (see **Figure 1A**) (77–80).

Experimental design: This assay consists of a transparent plastic or glass tube with removable caps on both ends. A treated filter paper is placed in the lining of one of the caps and mosquitoes are transferred into the tube. The tube is divided (not physically) into two parts representing a treated and an untreated side. Control experiments typically consist of filter paper on both sides that are treated with a solvent like acetone or ethanol.

Calculating repellency: The repellent efficacy of treatments can be measured by recording the location or behavior of mosquitoes at specific times throughout the experiment and comparing data from treatment and control experiments to determine repellency. The repellent efficacy of a treatment can be calculated as a repellent ratio (77) or as a percentage by using the following equation:


$$\frac{\left(\#\;of\;mosquitoes\;in\;untreated\;half\right)-\left(\#\;of\;mosquitoes\;in\;treated\;half\right)}{\left(Total\;\#\;of\;mosquitoes\right)}\;x\;100$$

Example from scientific literature: This type of apparatus has been used in several publications to measure mosquito repellency of new and/or established treatments (77–79). In 2006, Schultz and colleagues tested four treatments, catnip essential oil, Osage orange essential oil, elemol, and DEET at different concentrations using a version of a Tube assay, they called “Static-Air Repellency Chamber” (80). They performed two different experiments using this assay, one to screen for repellency in the treatments and another to test the residual repellency, how long a treatment repels mosquitoes. The first experiment tested each treatment at three different concentrations (0.1%, 0.5%, and 1%) using hexane as the solvent. They identified Osage orange essential oil to be an ineffective mosquito repellent at all concentrations tested. Next, they determined the residual repellency of the other three treatments (catnip essential oil, elemol, and DEET). They found that the repellency of elemol and DEET remained constant over 180 minutes, but catnip essential oil lost some repellency over time.

Variations: There are several different variations and ways to modify the experimental design of this type of assay to test unique and/or specific hypotheses. These variations include the orientation of the tube (horizontal or vertical), the dimensions (width and length), and material of the tube (opaque, glass, plastic) (77–80). Variations in tube assays are very easy to standardize and these assays can be used to test for repellency, attraction, olfactory desensitization, or toxicity of treatments. Another variation described by the World Health Organization (WHO) is called the Resting Site Choice Test, where mosquitoes are placed in a tube that has two cages on either end, one cage contains a treatment or pesticide and the other has no treatment or pesticide (92, 93).

**Strengths**:

- Flexibility for variations and modifications.
- Affordable.
- Practical for most laboratories.
- Great tool for initial screening of treatments.
- Easy to establish.

**Weaknesses**:

- Lack of mosquito attractants.
- Challenging to distinguish between spatial or contact repellency.
- Treatments are not applied to human skin, which may not reflect the real-world application of a mosquito repellent product.
- Needs additional assays to support findings.

#### 2.1.2 Close proximity response assays

Overview: The Close Proximity Response assay is another simple method that can be used to test mosquito repellency to a treatment (see **Figure 1B**) (50, 81).

Experimental design: This assay involves testing individual mosquitoes in a mesh-lined cage. The test mosquito is allowed to acclimate to its surroundings until it rests on one of the mesh walls of the cage for a specified amount of time. Upon this requirement being met, the wide portion of a modified 1000 µl pipette tip is held against the exterior side of the mesh where the mosquito is resting. The modified pipette tip contains a treated filter paper. The pipette tip is held up to the mosquito for a specified amount of time. If the mosquito flies away from the treatment within this time, the time of flight is recorded. The same mosquito can be used to test more than one treatment. Typically, a large number (>30) of individual mosquitoes are tested for each treatment. A filter paper treated with paraffin oil is used as a control.

Calculating repellency: The repellent efficacy of treatments is measured by recording the time points that a mosquito flew away from the treatment. The proportion or percent of mosquitoes that did not fly away from the treatment is calculated and compared to the control.

Example from scientific literature: This assay has been used in a couple scientific papers to screen for mosquito repellent treatments (50, 81). One of these studies was conducted in 2020, by Afify and Potter to test variations in the behavior of different mosquito species after exposure to the same treatments. In their experiment six established mosquito repellents (IR3535, DEET, Eugenol, Picaridin, PMD, and Lemongrass oil) were tested on three different species of mosquito (*Anopheles coluzzii*, *Aedes aegypti*, *Culex quinquefasciatus*). Afify and Potter found clear differences in the behavioral responses to treatments among the different mosquito species tested. They found that only lemongrass oil repelled all three species at a similar rate. PMD repelled *A. coluzzi* and *C. quinquefasciatus*, but only slightly repelled *A. aegypti*. Eugenol significantly repelled *A. aegypti* and slightly repelled *C. quinquefaciatus*. DEET slightly repelled *A. aegypti* and *C. quinquefasciatus*, but it did not repel *A. coluzzi*. IR3535 and picaridin did not show evidence for mosquito repellency in this assay.

Variations: Some variables that can be altered are the size of the cage and the concentration of treatments used. This assay could be used to test if mosquitoes develop an olfactory blindness to treatments that they are exposed to for a certain duration of time.

**Strengths**:

- Affordable.
- Practical for most laboratories.
- Great tool for initial screening of treatments.
- Tests for spatial repellency.
- Easy to establish.

**Weaknesses**:

- Lack of mosquito attractants.
- Cannot test for contact repellency.
- The presence of an experimenter’s hand and overall experimenter presence may add an unaccounted-for variable.
- The movement of the experimenter may cause mosquitoes to fly away which may be confused for repellency of the treatment.
- Requires several repeats to compensate for random mosquito flight or experimenter influence.
- Treatments are not applied to human skin, which may not reflect the real-world application of a mosquito repellent product.
- Needs additional assays to support findings.

#### 2.1.3 Excito-repellency test chamber assays

Overview: The Excito-Repellency Test Chamber (ER) assay is an effective technique to evaluate the repellency of a treatment by measuring the number of mosquitoes that escape from a treated chamber to an untreated one (see **Figure 1C**) (82–90).

Experimental design: This apparatus is a box-shaped chamber with an escape port that leads to an untreated receiving chamber. The main chamber contains treated fabric or paper. Mosquitoes are transferred into the main chamber and acclimatize for a specified amount of time before the escape port is opened. Mosquitoes can either escape from the main chamber into the receiving port or remain in the main chamber. At specified intervals, the number of escaped mosquitoes is recorded throughout the duration of the study. The treated paper or fabric is situated in the main chamber. It can be exposed to landings (direct contact) or covered to prevent landings. An untreated fabric or paper is used as a control.

Calculating repellency: The repellent efficacy of a treatment is measured by recording the number of mosquitoes that remained in the main chamber and the number of mosquitoes that escaped into the untreated receiving chamber at the end of each experiment. To calculate escape rates, the data can be analyzed using a Kaplan-Meier survival analysis where escaped mosquitoes are counted as deaths and remaining mosquitoes are survivals (88). Using this analysis, mosquito escape rates when exposed to treatments are calculated and this value is used to compare mosquito repellency between treatments.

Example from scientific literature: This type of assay has been developed and modified in several studies starting as early as 1973 (82). A standardized experimental design called the “Excito-repellency test” was developed in the early 90s (83–87, 89). An example of a publication that uses the ER assay comes from Boonyuan and colleagues (88). In this 2014 study, the repellent efficacy of essential oil extracts from five different plants (hairy basil, ginger, lemongrass, citronella grass, and plai) was tested. Each essential oil treatment was tested at different concentrations, 2.5%, 5%, and 10%, using ethanol as a solvent. Treatments were applied to filter paper. Boonyuan and colleagues tested each treatment in a 30-minute contact and a 30-minute non-contact experiment. They found the hairy basil essential oil extract to have the strongest repellent effect at a 2.5% concentration, followed by 5% lemongrass oil, 5% citronella oil, and 5% ginger oil.

Variations: A variation of the ER assay called the “High-Throughput Screening System” or HITSS, was developed by Grieco and collaborators in 2005 (91) and utilizes a cylinder-tube shaped apparatus instead of a box-shaped chamber. The experimental design is similar, mosquitoes are placed in a treated region and allowed to escape into an untreated area. The number of mosquitoes escaped or knocked down is recorded and the data is used to infer the repellency or pesticidal activity of treatments.

**Strengths**:

- Consistency in experimental design.
- Can test either contact or spatial repellency.

**Weaknesses**:

- Lack of mosquito attractants.
- May not be feasible for all labs.
- Treatments are not applied to human skin, which may not reflect the real-world application of a mosquito repellent product.
- Needs additional assays to support findings.

### 2.2 Repellency assays with an attractant source

These laboratory assays measure changes in mosquito host-seeking behavior towards an attractant source in the presence of a treatment. There are several different variations and methods used to test mosquito repellents that include an attractant component. The assays covered in this category have the following common elements in their experimental designs: an apparatus that contains test mosquitoes and a mosquito attractant located in conjunction with or proximal to a treatment.

Attractant sources used for these assays can range from, or be a combination of, synthetic odors, CO_2_, heat, animal hosts, or human volunteers. Mosquito repellent treatments, tested in this category of assays induce a reduction or complete mitigation of mosquito host-seeking behavior, displayed by either changes in mosquito location, mosquito landing, or mosquito biting and feeding, compared with controls.

A strength of assays that have an attractant component is the ability to gather compelling evidence on the mosquito-repelling properties of a treatment. If the treatment conveys repellency, the data collected from these assays generally show clear differences between the host-seeking behavior in mosquitoes exposed to a control or treatment. A general disadvantage of these assays is the wide variations in mosquito host-seeking behavior and the impact of variables that, at times, can be difficult to account for, predict, or control. Some of these variables include environmental variations such as time-of-day, lighting, temperature, humidity, and season. Others concern organismal variations, such as mosquito species, age, different stressors, larval and adult densities, and natural variations in attraction to different attractant sources. In these types of assays, it is crucial to frequently run control tests to assure that mosquitoes are actively host-seeking during and between experiments.

For this review, I split laboratory repellency assays with an attractant source into two groups: spatial repellency assays and contact repellency assays.

#### 2.2.1 Spatial repellency assays

Spatial repellency assays measure changes in mosquito location relative to an attractant source that is proximal to, or coated by, a treatment. In spatial repellency tests, mosquitoes do not directly contact the treatment and can either fly towards or away from it. These types of assays generally calculate the “reduction in mosquito attraction” relative to the mosquito attraction measured in a control. A treatment that does not repel mosquitoes will result in a relatively high number of mosquitoes flying towards an attractant source. While a treatment that is an effective mosquito repellent will have less or a complete reduction in the number of mosquitoes flying toward the attractant source.

Below are some common and useful assays that use an attractant source to measure the spatial repellent activity of treatments on mosquitoes (52, 94–113).

#### 2.2.2 Y-tube olfactometer assays

**Figure 2:**
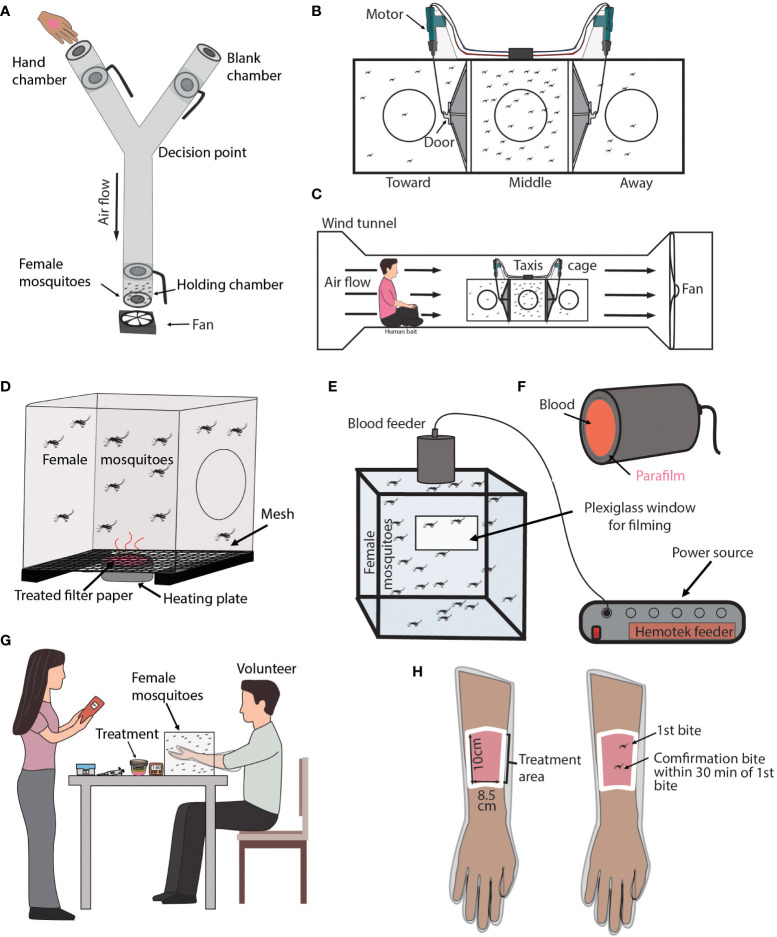
Laboratory repellency assays with an attractant source. **(A)** Diagram of Y-tube Olfactometer assay. Shown is a transparent Y-tube that is laid down horizontally with a small fan placed at the base of the “Y”. Each chamber has a small door that can be rotated open or close. The holding chamber contains acclimating mosquitoes. The pink circle on the hand represents a treatment. The location of the hand alternates between chambers for each replicate. **(B)** Diagram of a Taxis Cage. Shown are three cages connected by doors that can be opened or closed by the remote-controlled motor. Each cage is labeled either toward, middle, or away in relation to the volunteer’s location. **(C)** Diagram of a Taxis Cage located in a Wind Tunnel. Shown is a volunteer sitting near a Taxis Cage. The volunteer’s pink shirt represents a treatment. **(D)** Diagram of a Surface Landing assay. Shown is a mosquito-infested cage with a heated plate located underneath it. The pink area of the heated plate represents a treatment. **(E)** Diagram of a Feeding assay. Shown is a cage filled with mosquitoes. A plexiglass window is used to film mosquito feeding and behavior. A heated feeder filled with blood is located at the top of the cage. **(F)** Diagram of a blood feeder used in the Feeding assay. The red region represents the blood within the feeder and the parafilm is treated, indicated by the pink font. **(G)** Diagram of the Arm-In-Cage assay. Shown is a volunteer with their arm inserted in a mosquito-infested cage. **(H)** Diagram of the volunteer’s arm that is used in the Arm-In-Cage assay. Shown is a sleaved arm. The white border represents the cutout region where the volunteer’s skin is exposed to mosquitoes. The exposed skin is treated which is shown in pink.

Overview: The Y-tube Olfactometer assay evaluates the spatial repellency of a treatment by measuring the number of mosquitoes that fly toward an attractant in the presence or absence of a treatment (see **Figure 2A**) (52, 94–104).

Experimental design: This apparatus is a “Y” shaped tube with chambers at each end that can be opened or closed. A fan located at the base of the “Y” is used to create an airflow through the tube. Mosquitoes are transferred into a holding chamber located at the base of the “Y” and are given a specified amount of time to acclimate. A volunteer’s natural odors, body heat, and carbon dioxide are used as an attractant source for control and treatment experiments. The volunteer and treatment never come into direct contact with the test mosquitoes. For treatment experiments, the volunteer’s hand is either coated with the treatment or the volunteer holds a container containing the treatment in their palm. After the acclimation time, all chambers are opened, and the mosquitoes can fly throughout the apparatus. Mosquitoes can either remain in the holding chamber, fly toward the volunteer’s hand, or fly toward the blank chamber. After a specified amount of time, the chambers are closed, and the number and location of all mosquitoes are recorded. An untreated hand is used as a control.

Calculating repellency: The repellent efficacy of a treatment is measured by recording the number of mosquitoes in each location to calculate the percent attraction using the following equation:


$$\frac{\left(\#\;of\;mosquitoes\;located\;in\;the\;hand\;chamber\right)}{\left(Total\;\#\;of\;mosquitoes\right)}\;x\;100$$

Example from scientific literature: Since the last couple of decades, the Y-tube Olfactometer assay has become a staple test for measuring mosquito spatial repellency and attraction (94). The design of the Y-tube olfactometer apparatus has varied significantly, however the general experimental design has remained consistent. This assay has been frequently used in many scientific research papers (95–103) and has been recommended by the World Health Organization (WHO) in their “Guidelines for efficacy testing of spatial repellents” (52). To promote a more standardized apparatus and experimental design, the WHO published specifications on dimensions and shape for the Y-tube. Rodriguez and colleagues used these specifications in their study in 2015 to test seven commercially available mosquito repellents, a perfume, bath oil, and a vitamin B patch (104). In this study, a volunteer’s hand was treated and tested at initial treatment application, and 30-, 120-, and 240-minutes post-treatment. Rodriguez and colleagues tested all treatments on both *A. aegypti* and *A. albopictus*. They measured a 61% and a 41% mosquito attraction to the untreated volunteers’ hands with *A. aegypti* and *A. albopictus*, respectively. They found that of the commercially available repellents tested on *A. aegypti*, products containing DEET displayed a significant reduction in attraction at all time points tested. DEET-free products conferred various levels of reduction in attraction. They found no spatial mosquito repellency when testing the vitamin B patch.

Variations: Variables that can be modified in the Y-tube assay include the speed of airflow in the tube, the species of mosquito tested, the type of attractant source, and the duration of replicates. A similar assay to the Y-tube is the Uniport Olfactometer. The Uniport assay is a cylinder-shaped apparatus where mosquitoes are placed in one location and can move towards or away from an attractant source in the presence or absence of a treatment (81, 114). The major difference between these two assays is that in the Y-tube assay mosquitoes can make a decision between two branched chambers, this allows to address different questions such as competition between two treatments (115).

**Strengths:**

- Flexibility for variations and modifications.
- Presence of a mosquito attractant.
- Tests for spatial repellency.
- Can test one treatment or competition between two treatments.

**Weaknesses:**

- Variation in attractant sources.
- Cannot test for contact repellency.
- Needs frequent control testing.

#### 2.2.3 Taxis Cage assays

Overview: The typical Taxis Cage assay evaluates the spatial repellency of a treatment by recording the changes in mosquito location relative to the location of a human volunteer in the presence or absence of a treatment (see **Figure 2B**) (105–113).

Experimental design: The Taxis Cage is an apparatus, consisting of three boxy cages. Adjacent cages are connected by a port that can be opened or closed. When all ports are open, mosquitoes can move towards an attractant source that is typically located outside at a specified distance from the apparatus. The sides of each cage are mesh to allow for air flow throughout the taxis cage. The cage can be set up with a fan used to create an airflow through the cages or can be placed in a large wind tunnel environment (see **Figure 2C**). A volunteer’s natural odors, body heat, and carbon dioxide are used as an attractant source. The volunteer sits at a specified distance from the taxis cage. Mosquitoes are transferred to the center cage and are left to acclimate. After the acclimation period, the ports connecting all three cages are opened and the mosquitoes can travel between cages for the duration of the experiment. At the end of the experiment, the ports are closed and the numbers of mosquitoes in each cage are counted. Treatments tested in this type of study can be topical treatments or free-standing products meant for outdoor use, such as candles or incense burners. Treatments are applied to or placed in front of the volunteer. In the presence of an effective mosquito repellent treatment, less mosquitoes will fly toward the volunteer compared to the control, and vice versa. An untreated volunteer is used as a control.

Calculating repellency: The repellent efficacy of a treatment can be measured by calculating the percent of mosquitoes attracted to the volunteer in the presence or absence of a treatment by using the following equation:


$$\frac{\left(\#\;of\;mosquitoes\;located\;in\;cage\;closest\;to\;attractant\right)}{\left(Total\;\#\;of\;mosquitoes\right)}\;x\;100$$

Example from scientific literature: Using box-shaped apparatuses for olfactometer tests to identify mosquito attractants or repellents has been a prominent method over the past couple of decades. There have been several complex and thoroughly designed apparatuses that use this type of experimental design to test different treatments (105–109). However, more simplified designs have been developed and published (110–112). A current Taxis Cage was designed by Lorenz and colleagues in 2013. In their study, the movement of *Anopheles gambiae* mosquitoes was measured in response to three different attractant olfactory cues using the taxis cage (113). These olfactory cues were carbon dioxide, synthetic odor blend combined with carbon dioxide, or a human volunteer. Lorenz and colleagues performed two experiments using the taxis cage, one in a semi-field environment and one in an open field environment. The three cues were placed 20-, 50-, 70-, or 100-meters away from the taxis cage. They found that at a 20-meter distance all three cues significantly attracted approximately 60% of the mosquitoes tested. At a 50-meter distance the carbon dioxide, and the synthetic odor blend combined with carbon dioxide, attracted mosquitoes, but the human volunteer did not. At a 70-meter distance only the synthetic odor blend combined with carbon dioxide significantly attracted mosquitoes, and at 100-meters none of the olfactory cues attracted any mosquitoes.

Variations Some variations that can be applied to the Taxis Cage are the location the experiment is conducted in, the species and number of mosquitoes, and the distance of treatments to the Taxis Cage. An alternative assay that has a similar methodology is the WHO Tunnel Test, where a live animal is placed inside the apparatus serving as the attractant source (92, 93). This variant to the Taxis Cage is designed specifically to test insecticide-treated bed nets, where mosquitoes must cross through holes in a treated net to approach the attractant source.

**Strengths**:

- Can be placed in several different environments (a room with a fan attached, a wind tunnel, in a semi-field site, and in a field site).
- Presence of mosquito attractant.
- Tests for spatial repellency.
- Can test free-standing devices and mosquito repellent methods meant for outdoor-use.

**Weaknesses**:

- Variation in attractant sources.
- Cannot test for contact repellency.
- Needs frequent control testing.
- May not be feasible for all labs.

#### 2.2.4 Contact repellency assays

Contact repellency assays measure changes in mosquito host-seeking behavior relative to an attractant source that is proximal to, or coated by, a treatment. In contact repellency tests, mosquitoes can make direct contact with the treatment and can either initiate feeding behaviors like landing, probing, and engorging, or fly away from it. These types of assays generally calculate repellency by measuring either the time of or the number of landings, probing, or blood meals in the presence of a treatment compared to the control. A treatment that does not repel mosquitoes will have a relatively high number of mosquitoes quickly initiating feeding behaviors towards the attractant source. While a treatment that is an effective mosquito repellent will have less or a complete reduction in the number of mosquitoes conveying these behaviors toward the attractant source for a longer period of time.

Below are some common and useful assays that use an attractant source to measure the contact repellent activity of treatments on mosquitoes (41, 116–135).

#### 2.2.5 Surface landing assays

Overview: The Surface Landing assay evaluates the repellency of a treatment by measuring the number of mosquito landings on an attractive platform that has been treated (see **Figure 2D**) (116–119).

Experimental design: The typical apparatus is a mesh cage with a heated element and an attractive odor blend located on one of the cage walls. The heat element is usually set to 36-37°C. Mosquitoes are transferred into the cage and given time to acclimate. After the acclimation period, mosquito landings or probing on the heated element are recorded over time. Treatments are applied to the heated element, along with the attractive odor blends, either by using a treated fabric or paper.

Calculating repellency: Repellency is measured by recording the number of mosquito landings or probing on the mesh region of the cage directly above the heated platform. The number of landings or probing on this platform in the presence or absence of a treatment is compared to calculate mosquito repellency.

Example from scientific literature: The Surface Landing assay has been used to test mosquito repellents for a little over a decade (116–118, 136). In 2014, Menger and collaborators used this assay to compare nine prospective mosquito repellent compounds to DEET with *A. gambiae* (119). In their study, they used an odor mixture that mimics the scent of a human foot as an attractant source in addition to the heated platform and pulses of carbon dioxide. The odor mixture and treatments were applied to separate nylon strips. The nine different compounds that were tested in this study were 1-dodecanol (1DOD), 2-nonanone (2NON), 6-methyl-5-hepten-2-one (6MHO), 2,3-heptanedione (23HD), 2-phenylethanol (2PHE), eugenol (EUG), *δ*-decalactone (dDL), *δ*-undecalactone (dUDL) and linalool (LNL). They found that application of DEET, PMD, 2NON, 6MHO, LNL, dDL, and dUDL resulted in significantly less landings from *A. gambiae* mosquitoes compared to the controls (no treatment and ethanol treatment). dDL and dUDL had not been previously shown to repel mosquitoes, so Menger and colleagues continued their study focusing on these two compounds. They performed the same assay using *A. aegypti* mosquitoes and tested only DEET, PMD, dDL, and dUDL. They found again that dDL and dUDL performed similar to the positive controls (DEET and PMD).

Variations: This type of assay can vary in the location of heat elements and attractant sources inside or outside the apparatus, the type of attractant source used, the carrier materials, and the mosquito species (24, 108, 137).

**Strengths**:

- Presence of standardized mosquito attractant.
- Tests for contact repellency.
- Easy to establish.

**Weaknesses**:

- Treatments are not applied to human skin, which may not reflect the real-world application of a mosquito repellent product.
- Cannot test for spatial repellency.
- Needs frequent control testing.

#### 2.2.6 Feeding assays using artificial feeding systems

Overview: Feeding assays can evaluate the repellent efficacy of a treatment by measuring the feeding behavior and engorgement rates of mosquitoes in the presence of a treatment (see **Figures 2E, F**) (41, 120–123).

Experimental design: This design is straight forward, consisting of a test cage that has a heated feeding unit pressed alongside one of the cage’s meshed sides. A treated membrane or fabric is placed in between the feeder and the mesh. The feeder is typically filled with defibrinated blood and the number of mosquitoes that probe the feeder or engorge on blood is recorded.

Calculating repellency: Repellency is measured by recording mosquito probing or the rate of mosquito engorgement in the presence or absence of a treatment.

Example from scientific literature: Feeding assays are a common and frequently used assay in scientific research papers (120–123). This type of assay can be used to measure the efficacy of mosquito repellents or attractants, or to study mosquito behavior under different conditions. In 2019, Dennis and colleagues performed a study to further investigate the mechanism of action of DEET (41). They conducted two separate experiments. The first experiment was designed to test the anti-feedant properties of DEET by mixing DEET into the blood used in the artificial feeder. The second experiment was designed to test contact repellency of DEET by treating the membrane of the artificial feeder. They found that DEET mixed into blood strongly deterred mosquito blood feeding and that DEET applied to the feeding membrane completely deterred mosquito contact.

Variations: There are several variations that can be applied to these types of assays. These variations include the material that treatments are applied to, and the type of nutrients in the feeder, such as sugar solutions, Skitosnack, different types of blood (24, 138, 139). Other variations can be the status of the mosquitoes used (age, health, genetic modifications, species, etc.), and the treatment’s location in the apparatus. For instance, there is a variation to this type of assay where the treatment is located only at the perimeter of the feeding unit on a cylindrical shaped filter paper (140, 141).

**Strengths**:

- Alternative to using human volunteers.
- Flexibility for variations and modifications.
- Presence of standardized mosquito attractant.
- Tests for contact repellency.

**Weaknesses**:

- Membranes used on the feeders may be dissolved by certain treatments like essential oils.
- Treatments are not applied to human skin, which may not reflect the real-world application of a mosquito repellent product.
- Cannot test for spatial repellency.
- Needs frequent control testing.

#### 2.2.7 Arm-In-Cage assays

Overview: Arm-In-Cage assays evaluate the repellent efficacy of a treatment by measuring mosquito probing or landing on a human volunteer’s treated or untreated skin (see **Figures 2G, H**) (124–135).

Experimental design: This type of assay consists of a mosquito-infested cage in which a volunteer inserts their arm for a specified period. The volunteer’s arm is typically protected in an elbow-length glove that mosquitoes cannot penetrate, such as a plastic food-serving glove. The glove has a cutout at the inner-forearm region where the volunteer’s skin is exposed to host-seeking mosquitoes. The cutout is secured using fabric tape, and an exposed patch of skin is either treated or left untreated. The volunteer then places their arm in the mosquito-infested cage and continuously observes the exposed region of skin for mosquito landings or probing for a specified amount of time. An untreated control is used to confirm that the test mosquitoes are attracted to the volunteer’s arm.

Calculating repellency: Repellency can be measured by calculating the complete protection time (CPT) of a treatment. CPT is calculated by averaging the times of the first event (landing or probing) on the volunteer’s skin. The first event is the only data point used to calculate CPT. Typically, a second event is necessary for the first event to be validated and used. The second event must occur within a specified amount of time from the first event. Repellency can also be calculated in different metrics, such as by measuring percent bite protection for a treatment, using a “Biting Deterrence Index”, or measuring the minimum effective dosage of a treatment (136, 142).

Example from scientific literature: The Arm-In-Cage assay has been one of the most common and heavily relied-on assays to measure the repellent efficacy of treatments (124–131). This assay first took shape almost a century ago (132) and since then has been standardized and recommended by the Environmental Protection Agency (US-EPA) and the World Health Organization (133, 134). In 2023, Luker and colleagues used a version of this assay to test 20 active ingredients from the EPA’s Minimum Risk Pesticides List on *A. aegypti* mosquitoes (135, 143, 144). 19 of these active ingredients were oils or essential oils, and one was a terpene compound. They tested 10% emulsions in an organic lotion base for each treatment and found that of the 20 treatments tested, four provided CPTs of over 60 minutes. 10% clove oil protected from mosquito bites for almost 2 hours, while 10% Cinnamon Oil protected for about 1 hour and 30 minutes, and 10% 2-Phenylethyl Propionate and 10% Geraniol provided protection for about 1 hour.

Variations: There are many variations to the Arm-In-Cage assay in the current literature; besides the dimensions of the mosquito cage and the number of mosquitoes in the cage. These include using the hand or leg of a volunteer rather than the forearm, pressing the volunteer’s arm, hand, or leg against the mesh of the cage instead of inserting their arm, or using a treated cloth placed on the volunteer’s arm instead of applying treatment directly onto the volunteer’s skin (46, 81, 86, 89, 120, 145–150).

**Strengths**:

- Presence of mosquito attractant.
- Tests for contact repellency.
- Treatment used on human skin.

**Weaknesses**:

- Variation in volunteer attraction.
- Cannot test for spatial repellency.
- Needs frequent control testing.
- Uses human volunteers.

## 3 Conclusion and future directions

In this review, I covered several different laboratory methods that can be used to measure the repellent efficacy of specific treatments on mosquitoes (see **Table 1**). These assays were placed into two broad categories: assays without an attractant source and assays with an attractant source. While I covered many common and current laboratory methods and their variations, there are several more to be found in the published literature.

**Table 1:** Summary Table.

Attractant source?	Assay	Type of repellency tested	Variable measured	Human volunteers/live animals
No	Tube assay	General - cannot distinguish contact vs spatial	Mosquito location	No
No	Close Proximity Response assay	Spatial repellents	Time to mosquito flight	No
No	Excito-Repellency Test Chamber assay	Contact or spatial repellents	Mosquito escape rate	No
Yes	Y-tube Olfactometer assay	Spatial repellents	Percent mosquito attraction	Yes
Yes	Taxis Cage assay	Spatial repellents	Percent mosquito attraction	Yes
Yes	Surface Landing assay	Contact repellents	Mosquito landings	No
Yes	Feeding assay	Contact repellents	Mosquito probing or engorging	No
Yes	Arm-In-Cage assay	Contact repellents	Mosquito landing or probing	Yes

It is apparent that there are a multitude of ways to test the repellency of treatments on mosquitoes in a laboratory setting. Each method has its own strengths and weaknesses that I attempted to highlight throughout this review. Some can be used to screen multiple treatments in short periods of time, others are more refined and can be used to answer specific and unique questions. Assays can be used in conjunction to produce thorough and extensive research on the repellent efficacy of novel or established treatments.

Mosquito repellents can convey repellency in different ways, including spatially and/or through contact. Laboratory assays like the Excito-Repellency Test Chamber (non-contact version), Y-Tube, and Taxis cage are effective techniques to specifically measure for spatial repellent properties of a treatment, while the Surface Landing, Feeding, and Arm-In-Cage assays are effective to measure the contact repellent properties of a treatment. Other assays covered in this review can measure mosquito repellency to a treatment, but it may be difficult to distinguish between spatial and/or contact repellency when only using these assays alone.

This review also unveils how basic and straightforward many of these laboratory methods are. Nearly all of them are relatively low-tech with the most technical aspects being the optional use of cameras to record data or the use of computers to run statistical analyses.

Every year technology becomes more accessible, affordable, and user-friendly in the field of scientific research. In the next decade, I predict a surge of novel methods to test mosquito repellents that will revolutionize current screening methods. Some of the most promising technology is already prevalent in research such as video tracking. Video tracking has been used to graph arthropod behavior in an arena or container (151–153). Significant advancements in video tracking technology enables the development and implementation of specialized mosquito behavior studies (154). Another type of technology that can advance the study of mosquito repellents is the use of artificial intelligence (AI). AI can be used to predict novel mosquito repellent active ingredients based on molecular structures of known mosquito repellents and their targets (155). AI can also be used to collect and analyze large amounts of complex real-time data.

In conclusion, there are numerous established laboratory assays to test the repellent efficacy of treatments on mosquitoes and each has unique strengths and weaknesses. Due to technological advancements and new perspectives entering this field of research, there is continuous development of novel laboratory methods to test mosquito repellents. As time progresses, these novel methods may replace or improve the assays frequently used today.

## Author contributions

HL: Conceptualization, Investigation, Writing – original draft, Writing – review & editing.
